# Optimization of Sulfide Annealing Conditions for Ag_8_SnS_6_ Thin Films

**DOI:** 10.3390/ma16186289

**Published:** 2023-09-19

**Authors:** Ryuki Munekata, Tomohiro Uchimura, Hideaki Araki, Ayaka Kanai, Kunihiko Tanaka, Tomoichiro Okamoto, Yoji Akaki

**Affiliations:** 1 National Institute of Technology (KOSEN), Miyakonojo College, Miyakonojo 885-8567, Japan; munekatag4@miyakonojo.kosen-ac.jp; 2 Laboratory for Nanoelectronics and Spintronics, Research Institute of Electrical Communication, Tohoku University, Sendai 980-8576, Japan; uchimura.tomohiro.s8@dc.tohoku.ac.jp; 3 National Institute of Technology (KOSEN), Nagaoka College, Nagaoka 940-8532, Japan; h-araki@nagaoka-ct.ac.jp; 4 Department of Electrical, Electronics and Information Engineering Electronic Device and Light Wave Control Engineering Group, Nagaoka University of Technology, Nagaoka 940-2218, Japanokamoto@vos.nagaokaut.ac.jp (T.O.)

**Keywords:** Ag_8_SnS_6_ thin film, annealing condition, multistage annealing

## Abstract

Ag_8_SnS_6_ (ATS) has been reported to have a band gap of 1.33 eV and is expected to be a suitable material for the light-absorbing layers of compound thin-film solar cells. However, studies on solar cells that use ATS are currently lacking. The objective of this study is to obtain high-quality ATS thin films for the realization of compound thin-film solar cells using vacuum deposition and sulfide annealing. First, glass/SnS/Ag stacked precursors are prepared by vacuum deposition. Subsequently, they are converted to the ATS phase via sulfide annealing, and various process conditions, namely, annealing time, annealing temperature, and number of steps, are studied. By setting the heat treatment temperature at 550 °C and the heat treatment time at 60 min, a high-quality ATS thin film could be obtained. Multi-step heat treatment also produces thin films with nearly no segregation or voids.

## 1. Introduction

Solar cells have attracted considerable attention as a solution to current energy problems. However, current mainstream crystalline silicon solar cells have a low light-absorption coefficient and cannot be fabricated as thin films; moreover, their use is raising concerns regarding raw material depletion because they require large amounts of high-purity silicon. By contrast, Cu (In, Ga) Se_2_ (CIGS) solar cells have a high optical-absorption coefficient and can be fabricated as thin films; thus, they can be used to fabricate low-cost solar cells with relatively low concerns regarding raw material depletion. However, they use the rare metals Ga and In, and contain the toxic element Se, thereby presenting concerns regarding their environmental impacts. An environmentally friendly, earth-abundant, nontoxic, and inexpensive alternative compound is essential for the long-term commercialization of solar cells, and Cu_2_ZnSnS_4_ (CZTS) thin films have attracted attention as environmentally friendly, earth-abundant, and inexpensive alternatives considering these factors [1,2,3,4,5]. However, their disadvantage is poor crystal quality owing to their large number of elements. Therefore, Cu_2_SnS_3_ (CTS) thin-film solar cells have gained considerable attention [6,7,8]. CTS has a direct band-gap energy and a high absorption coefficient in the order of 10^4^ cm^–1^ [9,10,11,12,13]. However, the highest reported conversion efficiency of these cells is as low as 5.24% [14], possibly owing to the CTS band gap of 0.92–0.95 eV [15,16,17], which is far from the ideal band gap of 1.4 eV for the light-absorbing layer. Reportedly, a (Cu, Ag)_2_SnS_3_ thin film, in which Ag (a homologous element of Cu) is added to the CTS, is expected to improve the open-circuit voltage (V_OC_) and fill factor (FF) [18,19]. Furthermore, Ag_2_SnS_3_ in which Cu is replaced with Ag, has a band gap of 1.26 eV, which is close to the ideal value [20]. However, one drawback of Ag_2_SnS_3_ thin films is the difficulty in forming crystalline thin films [21]. Therefore, Ag_8_SnS_6_ (ATS) thin films with different composition ratios have been reported to have a band gap of 1.33 eV [22], which is closer to the ideal value. This suggests that ATS can theoretically achieve higher conversion efficiency than CTS. A comparison of surface SEM images of CIGS solar cells with power generation efficiencies of 16.3% [23] and 20.4% [24] confirms that the more efficient cells have larger grain sizes and no voids or cracks. Therefore, for high efficiency of solar cells, the necessary conditions for the thin film of the light-absorbing layer are the size of the grain and the absence of voids and cracks. ATS thin films have, up to now, been prepared using three different deposition methods: sputtering [22,25], spin coating [26], and electrodeposition [17]. However, all thin films have small grain sizes and numerous voids, which is not promising for highly efficient solar cell thin films [24]. The advantages of using the vacuum evaporation method include the possibility of high efficiency and low production cost. Previous CTS studies have shown that a flat, dense thin film is obtained when the concentration of H_2_S in the sulfide annealing gas mixture is increased to 2%, voids exist in the Cu-S compound when the concentration is changed to 4%, and heterophase and voids exist when the concentration is changed to 6% [27].

In this paper, the aim of our study is the optimization of sulfide annealing conditions for ATS thin films that are prepared by performing sulfide annealing of the fabricated stacked precursors via vacuum evaporation.

## 2. Materials and Methods

In this study, glass substrates of dimension 21 mm × 26 mm were used. The substrates were ultrasonically cleaned using a glass cleaning solution (30 min × 2 times), ultrapure water (30 min × 2 times), acetone (30 min × 2 times), and ethanol (60 min × 1 time). Subsequently, glass/SnS/Ag stacked precursors were fabricated via vacuum evaporation; thus, the ultimate vacuum was 5.0 × 10^−4^ Pa, and the substrate temperature during Ag and SnS deposition was room temperature and 300 °C, respectively. The film thickness of SnS (purity: 3N) and Ag (purity: 3N) was approximately 170 and 510 nm, respectively. The distance between the sample and the source was 20 cm.

Next, ATS thin films were deposited on glass substrates via sulfide annealing (heat treatment) in a mixture of H_2_S and N_2_ gas. Table 1 lists the sulfide annealing conditions for the experiments that investigated the effects of the annealing temperature on the thin films. Sample 1 was annealed at 450 °C for 20 min, sample 2 at 500 °C for 20 min, sample 3 at 550 °C for 20 min, sample 4 at 450 °C for 60 min, sample 5 at 500 °C for 60 min, and sample 6 at 550 °C for 60 min. Here, since the annealing time of the thin film that achieves the world’s highest conversion efficiency in CTS is 20 min, this time was also investigated in ATS [10]. Table 2 lists the sulfide annealing conditions for the experiments that investigated the effect of multi-step sulfide annealing on the thin films. Sample 7 underwent provisional annealing at 200 °C for 60 min and primary annealing at 450 °C for 60 min. Sample 8 was provisionally baked at 200 °C for 60 min, and primarily baked at 550 °C for 60 min. Sample 9 was provisionally fired at 200 °C for 60 min, then fired at 450 °C for 60 min, and finally fired at 550 °C for 60 min. Evidently from Table 1 and Table 2, the rates of temperature increases and decreases were not specifically controlled.

The prepared samples were analyzed by X-ray diffraction (XRD) to investigate their crystal structure. Elemental mapping and compositional analysis were performed by energy-dispersive X-ray spectroscopy (EDS; Japan BRUKER QUANTAX Flat QUAD 5060) at an acceleration voltage of 15 kV. The surface topography was examined using scanning electron microscopy (SEM; Japan Hitachi SU8020).

## 3. Results and Discussion

### 3.1. Effect of Annealing Temperature and Time on Thin Films 

**Figure 1:**
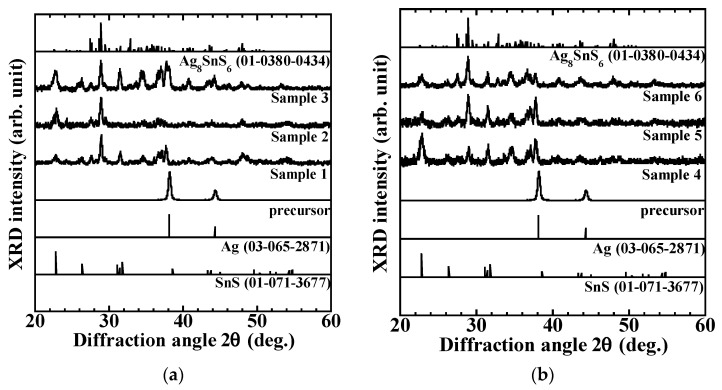
X-ray diffraction results for samples 1–6: (**a**) sample 1 (450 °C, 20 min), sample 2 (500 °C, 20 min), sample 3 (550 °C, 20 min); (**b**) sample 4 (450 °C, 60 min), sample 5 (500 °C, 60 min), sample 6 (550 °C, 60 min).

Figure 1 shows the XRD patterns of samples 1–6 prepared by performing sulfide annealing at different temperatures and times: the glass/SnS/Ag stacked precursors show only diffraction peaks at 38.12° and 44.30°. They are well identified with (111) and (200) diffraction lines for silver, respectively. No diffraction peaks attributed to SnS crystals were observed. This may be because the amount of deposited Ag was significantly larger than that of SnS, and Ag was present in the upper layer of the stack. Almost all samples showed a diffraction peak at 28.94° after sulfide annealing. This is well identified with the (022) diffraction line of the ATS crystal. Therefore, ATS crystals can be obtained by fabricating stacked precursors using vacuum deposition and sulfide annealing under the experimental conditions. However, samples 2–4 showed a strong diffraction peak at 22.82°. This is well identified with the (110) diffraction line of SnS crystal. This may be because of the lack of chemical reactions caused by the short annealing time and low annealing temperature. 

The lack of strong diffraction peaks attributed to the SnS crystals in sample 1 is considered to be a consequence of the low annealing temperature and short annealing time, which did not allow for good chemical reactions, thereby resulting in the formation of ATS in the upper layers and leaving unreacted SnS in the lower layers. Although there are some differences in peak intensities in the measurement results for diffraction angles of 30° to 40°, all peak positions are consistent with the PDF data. Therefore, we believe that the orientation state of the crystals after heat treatment changes depending on the state of the atoms and molecules in the precursor film and the heat treatment conditions. In sample 6, wherein sulfide annealing was performed at an annealing temperature of 550 °C for 60 min, the diffraction peaks attributed to ATS crystals became dominant, and the diffraction peaks attributed to SnS crystals became weaker. Therefore, sulfide annealing must be performed at an annealing temperature of 550 °C or higher and with an annealing time of 60 min or longer to obtain high-quality ATS thin films. Compared with the XRD patterns of ATS thin films obtained by sputtering, spin coating, and electrodeposition, the diffraction peaks attributed to ATS crystals were dominant, and thin films with weak unknown peaks were obtained, thus indicating that high-quality thin films, expected to be absorbing layers in solar cells, were obtained [17,22,25,26].

**Figure 2:**
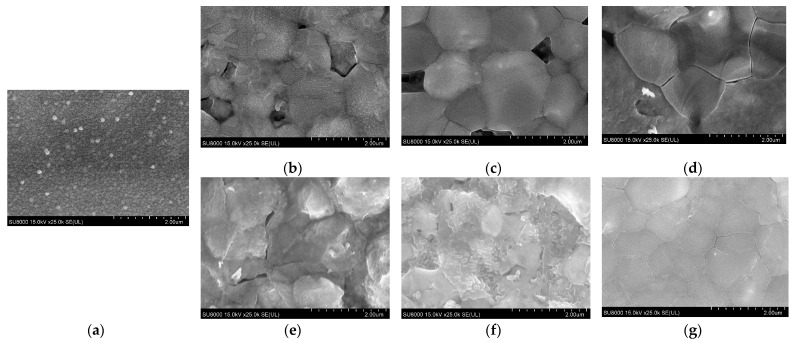
SEM images of sample surface observed using SEM: (**a**) precursor; (**b**) sample 1 (450 °C, 20 min); (**c**) sample 2 (500 °C, 20 min); (**d**) sample 3 (550 °C, 20 min); (**e**) sample 4 (450 °C, 60 min); (**f**) sample 5 (500 °C, 60 min); (**g**) sample 6 (550 °C, 60 min).

Figure 2 shows the SEM images of samples 1–6 prepared by performing sulfide annealing at different temperatures and times. On the surface of the glass/SnS/Ag stacked precursors, a uniform grain size of approximately 0.1 μm was observed, whereas in samples 1–3 after 20 min of sulfide annealing, the grain size increased to approximately 2.3 μm, but large voids and cracks were observed. In samples 4–6 after 60 min of sulfide annealing, the grain size increased, and the number of voids and cracks decreased as the annealing temperature increased. In sample 6, which was sulfide annealed at 550 °C for 60 min, cracks remained, but voids disappeared, and an ATS thin film with a maximum grain size of approximately 1.5 μm was obtained. Therefore, to obtain a flat ATS thin film, sulfurization needs to be performed at an annealing temperature of 550 °C or higher and with an annealing time of 60 min or longer. Compared with the SEM images of ATS thin films obtained by the sputtering, spin-coating, and electrodeposition methods, the grain size was large, no voids existed, and a flat surface was obtained, thus indicating that high-quality thin films, expected to be absorbing layers in solar cells, were obtained [17,22,25,26].

**Figure 3:**
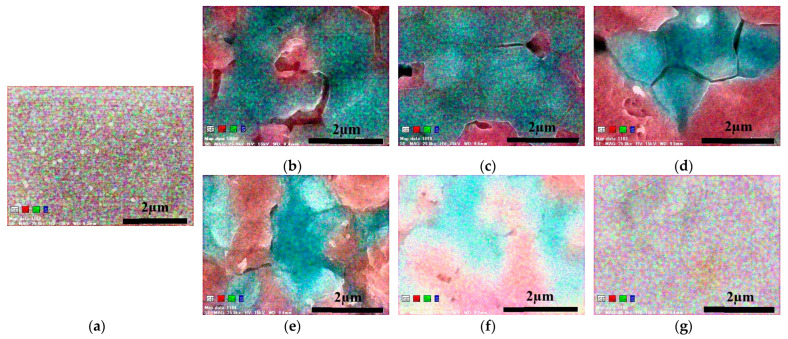
Elemental mapping of sample surface observed using EDS: (**a**) precursor; (**b**) sample 1 (450 °C, 20 min); (**c**) sample 2 (500 °C, 20 min); (**d**) sample 3 (550 °C, 20 min); (**e**) sample 4 (450 °C, 60 min); (**f**) sample 5 (500 °C, 60 min); (**g**) sample 6 (550 °C, 60 min).

Figure 3 shows the EDS surface elemental mapping images of samples 1–6 prepared by sulfide annealing at different temperatures and times. Here, red represents Ag, green represents Sn, and blue represents S elements; Ag, Sn, and S are uniformly distributed in the glass/SnS/Ag stacked precursor. Further, samples 1–3, which were sulfide-annealed for 20 min, were segregated as Ag (red) and Sn–S (green–blue). In samples 4–6, which were sulfide-annealed for 60 min, the elements became increasingly mixed as the annealing temperature increased. Furthermore, no segregation of Ag, Sn, or S was observed in sample 6, which was annealed at 550 °C for 60 min, thus suggesting the formation of an Ag–Sn–S compound. Therefore, sulfide annealing must be performed at an annealing temperature of 550 °C or higher and with an annealing time of 60 min or longer to obtain a single-phase ATS thin film.

**Figure 4:**
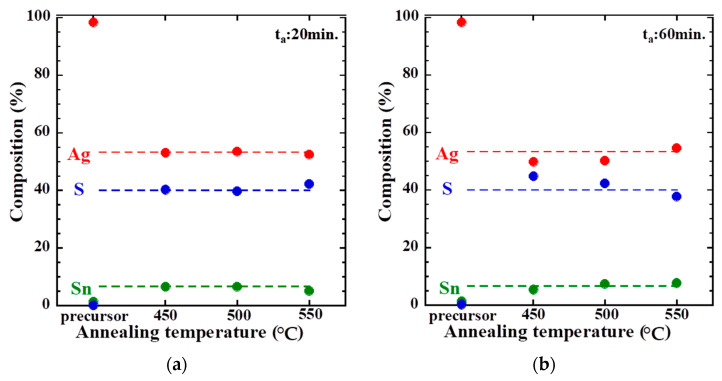
Compositional analysis results for samples 1–6: (**a**) precursor, sample 1 (450 °C), sample 2 (500 °C), and sample 3 (550 °C); (**b**) precursor, sample 4 (450 °C), sample 5 (500 °C), and sample 6 (550 °C).

Figure 4 shows the results of the composition analysis of samples 1–6 prepared via sulfide annealing at different temperatures and times. Here, the dotted line shows the stoichiometric composition. ATS has Ag:Sn:S = 8:1:6, thus resulting in 53.3% Ag, 6.7% Sn, and 40% S, whereas in the glass/SnS/Ag stacked precursors, Ag accounts for approximately 100%. In the XRD pattern, the glass/SnS/Ag stacked precursor resulted in Ag accounting for approximately 100% of the total. This is because the amount of Ag deposited is significantly higher than that of SnS and because Ag is in the upper layer of the laminate. In samples 1–3, wherein sulfide annealing was performed with an annealing time of 20 min, the results were nearly in accordance with the stoichiometric composition for samples at annealing temperatures of 450 and 500 °C. However, the sample at an annealing temperature of 550 °C had less Ag and Sn and more S compared with the stoichiometric composition. This may be attributed to excessive S compounding because of the higher annealing temperature. This is considered to have resulted in the relatively low composition ratio of Ag to S. In samples 4–6, wherein sulfide annealing was performed at an annealing time of 60 min, a trend of more Ag and Sn but less S with increasing annealing temperature was observed. In the samples with annealing temperatures of 450 and 500 °C, the results showed less Ag and Sn but more S compared with the stoichiometric composition, which is similar to the results obtained with an annealing time of 20 min and at an annealing temperature of 550 °C. This may be attributed to excessive S compounding because of the long annealing time. Although the composition ratio of Ag and Sn should gradually decrease and the amount of Sn should increase, the opposite results were obtained. This is because of the formation of SnS_2_ during sulfide annealing and its re-evaporation owing to the high annealing temperature and long annealing time. This is considered to have reduced the composition ratio of S and increased the composition ratio of Ag and S.

Although a high-quality thin film without voids was obtained by shortening the annealing time for CTS, a high-quality thin film without voids was obtained by annealing for 60 min, which is a lengthy duration for ATS in this experiment. This suggests that the ATS is less prone to chemical reactions compared with CTS. Another basis for this consideration is that the optimum annealing temperatures for obtaining high-quality thin films of CTS and ATS are 450 and 550 °C, respectively. Thus, regarding the annealing time and annealing temperature, annealing at 550 °C for 60 min (sample 6) produces the highest quality thin film, as evidenced by the results of XRD, surface morphology, and elemental mapping. However, based on XRD and composition analysis, certain problems existed; for example, the SnS heterophase and stoichiometric composition of SnS was not in accordance with the stoichiometric composition because of re-evaporation of SnS_2_ under this sulfide annealing condition. Therefore, further research is required in the future.

### 3.2. Effect of Multi-Step Annealing on Thin Films

**Figure 5:**
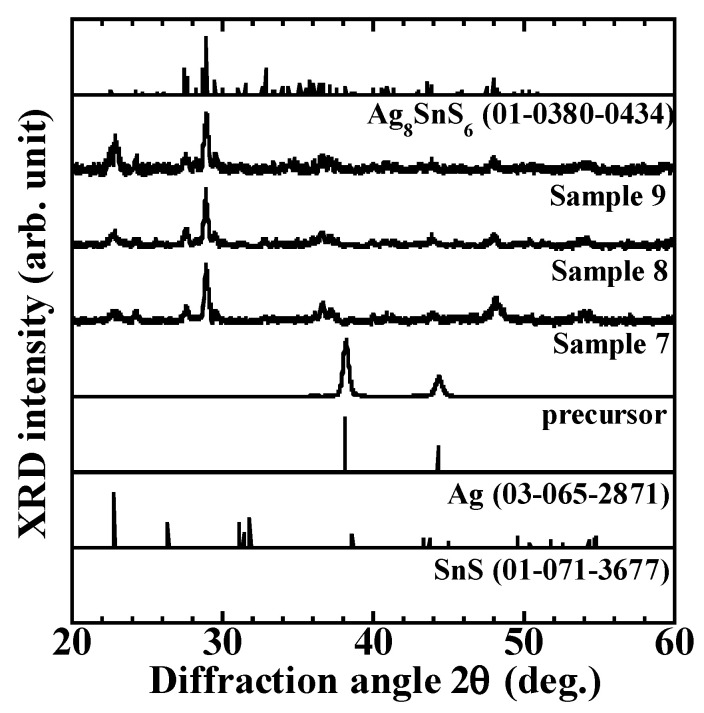
X-ray diffraction results for sample 7 (200 °C, 450 °C, 60 min), sample 8 (200 °C, 550 °C, 60 min), and sample 9 (200 °C, 450 °C, 550 °C, 60 min).

Figure 5 shows the XRD patterns of samples 1–6 prepared by sulfide annealing at different temperatures and times: the glass/SnS/Ag stacked precursors show only diffraction peaks at 38.12° and 44.30°. They are well identified with (111), and (200) diffraction lines for silver, respectively. No diffraction peaks attributed to SnS crystals were observed. This may be because of the amount of Ag deposited was considerably larger than that of SnS, and because Ag was in the upper layer of the stack. Almost all samples showed a diffraction peak at 28.94° after sulfide annealing. This is well identified with the (022) diffraction line of the ATS crystal. Therefore, ATS crystals can be obtained by fabricating stacked precursors using vacuum deposition and sulfide annealing under the experimental conditions. However, sample 9, which was fabricated after three steps of sulfide annealing, shows a strong diffraction peak at 22.82°. This is in good agreement with the (110) diffraction line of the SnS crystal. This may be because in samples 7 and 8 prepared by two-step sulfide annealing, SnS was present in the lower layer of the ATS thin film and appeared in the upper layer of the film because of the re-evaporation of SnS or SnS_2_ after a long annealing time of 3 h. The presence of SnS in the lower layer of the ATS thin film is because of a weak chemical reaction that occurred in the upper layer during the first step of the multi-step sulfide annealing process at 150 °C for 60 min; this resulted in the formation of the ATS thin film in the upper layer only, leaving SnS in the lower layer. SnS remained in the lower layer. Therefore, to obtain high-quality ATS thin film using multi-step sulfide annealing, the annealing time must be shortened during sulfide annealing at low temperature in the early stage of the process because 60 min duration is extremely long. Compared with the XRD patterns of ATS thin films prepared by sputtering, spin-coating, and electrodeposition methods, as well as with the XRD patterns of ATS thin films prepared using the annealing temperature and time in this study, the diffraction peaks attributed to the ATS crystal were dominant, and the unknown peaks were weak, thus indicating that high-quality thin films, expected to be absorption layers in solar cells, were obtained [17,22,25,26].

**Figure 6:**
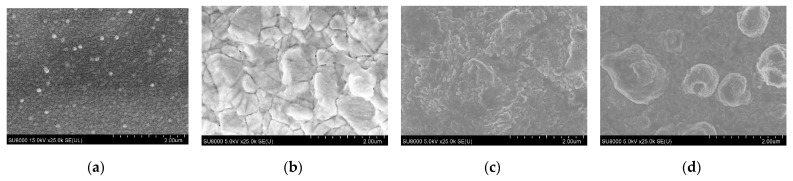
SEM image of sample surface observed using SEM: (**a**) precursor; (**b**) sample 7 (200 °C, 450 °C, 60 min); (**c**) sample 8 (200 °C, 550 °C, 20 min); (**d**) sample 9 (200 °C, 450 °C, 550 °C, 20 min).

Figure 6 shows SEM images of samples 7–9 prepared by performing multi-step sulfide annealing; evidently, a uniform grain size of approximately 0.1 µm was observed on the surface of the glass/SnS/Ag stacked precursors. Sample 7 showed crystal growth after multi-step sulfide annealing, essentially obtaining a grain size of approximately 0.8 µm. However, although crystallinity was observed, multiple voids and cracks were present. No voids or cracks were observed in samples 8 and 9, but crystallinity could not be confirmed. It was found that the crystallinity of the thin films decreased as the annealing temperature and time increased. Therefore, the annealing temperature and time must be optimized to obtain high-quality ATS thin films using multi-step sulfide annealing.

**Figure 7:**
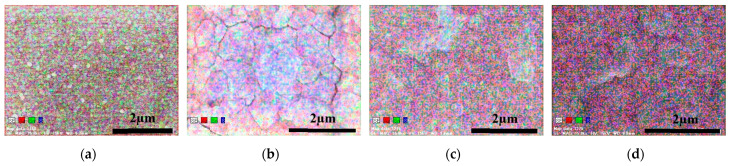
Elemental mapping of sample surface observed using EDS: (**a**) precursor; (**b**) sample 7 (200 °C, 450 °C, 60 min); (**c**) sample 8 (200 °C, 550 °C, 20 min); (**d**) sample 9 (200 °C, 450 °C, 550 °C, 20 min).

Figure 7 shows the EDS surface elemental mapping images of samples 7–9 prepared by performing multi-step sulfide annealing; here, red represents Ag, green represents Sn, and blue represents S elements. Evidently, Ag, Sn, and S were uniformly distributed in the glass/SnS/Ag stacked precursors. Minimal segregation was observed in the samples under all the sulfide annealing conditions. Therefore, multi-step sulfide annealing is suitable for obtaining thin films without segregation. Furthermore, when sulfidation annealing was performed three times, the overall color became strongly reddish-purple, thus suggesting the formation of the ATS compound; because ATS is Ag (red):Sn (green):S (blue) = 8:1:6, the strong reddish-purple color represents ATS. Therefore, multi-step sulfide annealing is considered suitable for obtaining high-quality ATS thin films without segregation.

**Figure 8:**
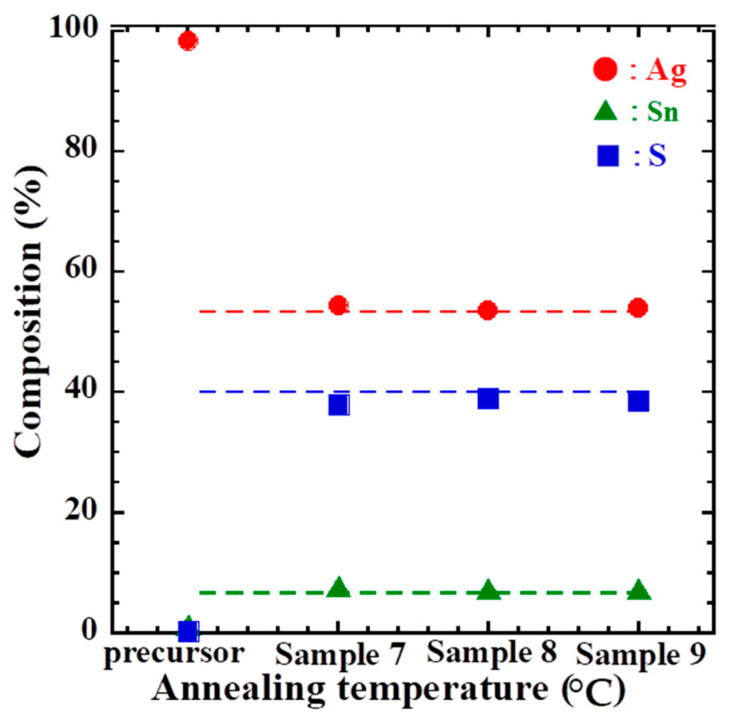
Compositional analysis results for sample 7 (200 °C, 450 °C, 60 min), sample 8 (200 °C, 550 °C, 60 min), and sample 9 (200 °C, 450 °C, 550 °C, 60 min).

Figure 8 shows the results of the compositional analysis of samples 7–9 prepared by performing multi-step sulfide annealing; here, the dotted line indicates the stoichiometric composition. ATS has Ag:Sn:S = 8:1:6, thus resulting in 53.3% Ag, 6.7% Sn, and 40% S. In the glass/SnS/Ag stacked precursor, Ag accounts for approximately 100%. As with the XRD pattern, this is likely because of the significantly larger amount of Ag deposited than that of SnS and because Ag is in the upper layer of the laminate. None of the samples 7–9 produced by performing multi-step sulfide annealing were in stoichiometric composition, and no law of small errors or sulfide annealing conditions were identified. Therefore, multi-step thermal sulfide annealing is suitable for obtaining high-quality ATS thin films with stoichiometric compositions.

In the multi-step annealing study, the crystallinity was considered to have deteriorated because of the long annealing time. Possibly, low-temperature annealing at 200 °C caused a slight chemical reaction only in the upper layers of the laminated precursor; therefore, annealing at 450 and 550 °C (i.e., the main baking process) did not cause a sufficient chemical reaction. In addition, the diffraction peak of SnS was stronger than that of sample 9, which underwent three-step annealing, whereas it was weaker in samples 7 and 8. This was also because the low-temperature annealing caused a slight chemical reaction only in the upper layer of the laminated precursor, thereby leaving SnS in the lower layer, and the main firing of samples 7 and 8 left SnS in the lower layer. However, because the main firing was conducted for twice as long as that in sample 9, a chemical reaction occurred with the upper layer but ended halfway through. However, as in sample 9, a chemical reaction with the upper layer occurred but ended in the middle of the reaction, which may have caused the SnS to remain. Therefore, multi-step annealing with low-temperature annealing before the main baking process prevents chemical reactions from occurring and produces a void-free thin film. In the future, we will consider reducing the annealing time at each step to suppress chemical reactions during low-temperature annealing before the main sintering to enable sufficient crystal growth during the main sintering.

## 4. Conclusions

In this study on annealing temperature and time, the highest quality Ag_8_SnS_6_ thin film was obtained at a 2% H_2_S concentration, at an annealing temperature of 550 °C, and with an annealing time of 60 min. This suggests that Ag_8_SnS_6_ is more difficult to chemically react than Cu_2_SnS_3_. Further studies are planned regarding annealing for longer times and at higher temperatures. We also believe that this satisfies the surface morphology of the thin film, which leads to higher efficiency of solar cells. The investigation of multi-step sulfide annealing explored the disappearance of voids in the thin film. Furthermore, multi-step sulfide annealing is suitable for the formation of stoichiometric thin films without the segregation of elements. However, this causes a deterioration in crystallinity, and we plan to examine the annealing time as a possible cause. It is possible to suppress chemical reactions that occur on the surface of the glass/SnS/Ag precursor by performing low-temperature annealing prior to the main sintering; therefore, sufficient crystal growth can be achieved during the main sintering. In addition, we plan to study the possibility of the slow annealing of sulfides by decelerating the rate of the temperature increase instead of performing sulfide annealing in stages because this may also be effective.

## Figures and Tables

**Table 1:** Effect of annealing temperature and time on thin films.

Name	Annealing Temperature	Annealing Time
sample 1	450 °C	20 min
sample 2	500 °C	
sample 3	550 °C	
sample 4	450 °C	60 min
sample 5	500 °C	
sample 6	550 °C	

**Table 2:** Effect of multi-step sulfide annealing on thin films.

Name	Annealing Conditions		
	1st Step	2nd Step	3rd Step
sample 7	200 °C, 60 min	450 °C, 60 min	
sample 8	200 °C, 60 min	550 °C, 60 min	
sample 9	200 °C, 60 min	450 °C, 60 min	550 °C, 60 min

## Data Availability

Not applicable.
